# Gap Junction Intercellular Communication in the Carcinogenesis Hallmarks: Is This a Phenomenon or Epiphenomenon?

**DOI:** 10.3390/cells8080896

**Published:** 2019-08-14

**Authors:** Roberto Zefferino, Claudia Piccoli, Sante Di Gioia, Nazzareno Capitanio, Massimo Conese

**Affiliations:** 1 Department of Medical and Surgical Sciences, University of Foggia, Via L. Pinto, 1-71122 Foggia, Italy; 2 Department of Clinical and Experimental Medicine, University of Foggia, Via L. Pinto, 1-71122 Foggia, Italy

**Keywords:** cancer, hallmark, connexins, microenvironment, inflammation, metastasis, angiogenesis, stem cells

## Abstract

If occupational tumors are excluded, cancer causes are largely unknown. Therefore, it appeared useful to work out a theory explaining the complexity of this disease. More than fifty years ago the first demonstration that cells communicate with each other by exchanging ions or small molecules through the participation of connexins (Cxs) forming Gap Junctions (GJs) occurred. Then the involvement of GJ Intercellular Communication (GJIC) in numerous physiological cellular functions, especially in proliferation control, was proven and accounts for the growing attention elicited in the field of carcinogenesis. The aim of the present paper is to verify and discuss the role of Cxs, GJs, and GJIC in cancer hallmarks, pointing on the different involved mechanisms in the context of the multi-step theory of carcinogenesis. Functional GJIC acts both as a tumor suppressor and as a tumor enhancer in the metastatic stage. On the contrary, lost or non-functional GJs allow the uncontrolled proliferation of stem/progenitor initiated cells. Thus, GJIC plays a key role in many biological phenomena or epiphenomena related to cancer. Depending on this complexity, GJIC can be considered a tumor suppressor in controlling cell proliferation or a cancer ally, with possible preventive or therapeutic implications in both cases.

## 1. Introduction

Cancer is a very complex disease. Although it is the second leading cause of death worldwide [1], its causes are largely unknown, if occupational tumors are excluded. Cancer risk is increased by inherited and acquired causes, therefore it is surely a multifactorial disease [2,3]. The first scientific attention focused much more on genetic aspects (genotoxic effects); later, epigenetic and metagenetic effects were also considered, and there is not agreement in the scientific community to-date on the different importance of genetic, epigenetic, and metagenetic factors [4,5,6].

Cell–cell communication is fundamental for maintaining tissue homeostasis, allowing precise signaling in response to both external and internal stimuli. These integral communication mechanisms, including gap junction intercellular communication (GJIC), are necessary for cells either to remain in quiescence or undergo proliferation, differentiation, or apoptosis. Thus, it is no surprise that defects in GJIC will result in impaired cell homeostasis and likely lead to the development of cancer [7,8]. The paradigm of GJIC involvement in cancer has been put forward since the 1960s, and since then, has been expanded and challenged.

Early observations showed that not all the carcinogens induce DNA damage, inhibit repair of DNA damage, or directly cause mutations, and not a few agents were shown to contribute to the promotion phase of carcinogenesis. These observations led to the notion that “epigenetic”, or more generally speaking, “metagenetic” mechanisms contribute to the promotion phase of carcinogenesis. Chemicals, such as 12-O-tetradecanoyl-phorbol-13-acetate (TPA), dichlorodiphenyltrichloroethane (DDT), 2,3,7,8-Tetrachlorodibenzodioxin (TCDD), polybrominated biphenyls (PBBs), polychlorinated biphenyls (PCBs), pentachlorophenol (PCP), phthalates, phenobarbital, and so on, which are not mutagenic and which do not “initiate” carcinogenesis, are good tumor promoters [9,10,11,12,13,14,15]. Interestingly, all these agents can induce oxidative stress and mitogenesis of initiated cells without killing them. Both of these processes (mitogenesis and apoptosis) require inhibited GJIC [12,16] and appear to be the cellular mechanism of tumor promotion [7,15]. Although various tumor promoters and many tested oncogenes inhibit GJIC, reversibly or stably, respectively, they do so via multiple biochemical mechanism at threshold levels [17]. In addition, the promotion process can be elicited by surgery, solid particles, or growth hormones, and cell death inducing compensatory hyperplasia (or chronic inflammation) [18].

The goal of this article is to discuss the hallmarks of cancer and verify, in this context, the role played by the GJIC with the aim to understand if it may be considered a phenomenon or epiphenomenon. First of all, it could be useful to specify the terms:

1. Hallmarks of cancer are: “acquired functional capabilities that allow cancer cells to survive, proliferate, and disseminate; these functions are acquired in different tumor types via distinct mechanisms and at various times during the course of multistep tumorigenesis” [19];
2. GJIC is the major mechanism used by biological systems enabling cells to work in an integrate way;
3. Epiphenomenon is a phenomenon that occurs contemporary to another but is not related to it [20].

### Gap Junctions, Hemichannels, and Connexins

Gap junctions (GJs) consist of aggregates of transmembrane hemichannels (or connexons) that dock to similar connexons on the neighboring cell with the intercellular distance estimated between 2–3 nm. While hemichannels are known to exhibit a function per se, including uptake and release of small molecules and passage of current [21], GJs allow small ions and molecules up to 1200 Daltons, including ions, amino acids, nucleotides, metabolites, and secondary messengers (e.g., calcium, glucose, cAMP, cGMP, IP3), to pass from one cell to the other [22]. Furthermore, the transfer of small interference RNAs between adjacent cells through GJ was demonstrated to be possible [23].

The connexons are formed by hexameric oligomers of transmembrane proteins, the connexins (Cxs). In humans, 21 members of this protein were described and named “Cx” followed by a number indicating their molecular mass ranging between 23 and 62 kDa [24]. The expression and distribution of different Cx isoforms is tissue and cell-type specific. Six identical Cxs oligomerize to form a homomeric connexon, and a connexon with more than one type of Cxs is called a heteromeric connexon. Two identical homomeric connexons dock head-to-head together to form a homomeric homotypic GJ channel, whereas two different homomeric connexons dock head-to-head together to form a homomeric heterotypic GJ channel. Two heteromeric connexons will form the homomeric heterotypic GJ channel even if they are identical. The assembly of such a variety of connexons in the GJ forms unique channels with specific permeability properties [25]. However, the functional meaning of this variety remains a conundrum. Along with Cxs, a family of structurally-related proteins, named pannexins, has been identified in vertebrates, including humans [26]. Pannexins share identical folding with Cxs and form oligomeric channels in the membrane. However, their role in forming cellular GJs is debated [27].

The crystallographic structure of the Cx26 GJ at 3.5 Å resolution has been reported [28] and provides essential information. As predicted from sequence analysis the connexin spans the membrane with a bundle of four alpha-helices interconnected by two extracellular loops and a cytoplasmic loop. A pair of alpha helices contribute to the internal wall of the GJ channel, while the other two face the hydrophobic membrane environment. The N and C terminus/domains are toward the cytoplasm. While the length and sequence of the four-helices is largely conserved among the different Cxs, the major differences in the extension and sequence are in the extra- and intracellular loops and in the N and C termini. This accounts for subtle or substantial differential functional properties or binding to effectors, or both. The overall structure of the GJs is featured by a positively charged entrance, a funnel, a negatively charged transmembrane pathway, and an extracellular cavity [28]. The funnel is determined by the six amino-terminal domains (for each connexon) lining the wall of the channel, thus determining the molecular size restriction at the channel entrance [28]. The published atomic structure of the Cx26 GJ corresponds to the open configuration. Recently, a stable open-state conformation was reported for Cx46/50 by single particle CryoEM analysis [29]. Nevertheless, a number of functional and mutational analyses suggest that a major structural determinant in tuning the open-closed transition involves a conformational change of the N-terminus domain [29,30]. In addition to the membrane potential, which electrically gates the GJ channel, several internal molecules are thought to be involved in the GJ modulation (e.g., Ca^2+^, H^+^, cyclic AMP, reactive oxygen/nitrogen species).

At the ultrastructural level, GJs are organized as hexagonally-structured plaque in freeze-fracture replicas with particle-rich areas (P-face) and areas with pits (E-face) [31,32,33]. As morphology is related to the function of cell–cell communication and, since, as we shall outline below, cancerous cells undergo changes in Cx expression, these are likely to be reflected in morphological changes of GJs [34]. GJs show a high plasticity (permanent assembly and turnover of particles) and, therefore, can be considered highly flexible components operating at the level of the regulation of cell proliferation and differentiation, as well as the maintenance of homeostasis. Key regulatory pathways occur with phosphorylation/dephosphorylation of Cxs, mainly at the C-terminal domain. Most of the following data refer to Cx43, being more widespread than other Cxs that are significantly expressed only in a few tissues. Phosphorylation determines disassembly and internalization of the GJ, due to the disengagement of Cxs from the GJ, whereas unphosphorylated proteins remain in GJ [35]. Several kinases, including protein kinase A, protein kinase C (PKC), p34(cdc2)/cyclin B kinase, casein kinase 1, mitogen-activated protein kinase (MAPK), and pp60(src) kinase, have been implicated in the phosphorylation of the C-terminal region of Cxs, regulating a variety of connexin processes, such as trafficking to membranes, assembly, degradation, and gating of functional GJ channels [35,36,37,38].

**Figure 1:**
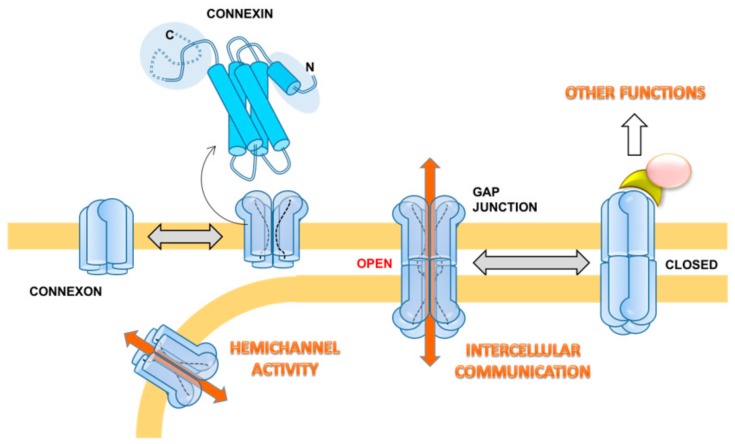
Schematic overview of the structural and functional features of connexins (Cxs). The folding of Cx is shown as inferred from the crystallographic structure of the Cx26 gap junction (GJ). The exameric connexons are shown in closed and open conformation, leading in the latter case to the hemichannel activity and GJ intercellular communication (GJIC). Non-GJIC or hemichannel related function of Cxs is also shown as resulting from interaction with cytosolic effectors.

Besides cell–cell communication, non-docked solitary connexons can have a hemichannel activity. This hands out the physiological extracellular release of different signaling molecules (e.g., ATP, glutamate, NAD^+^, PGE_2_, NO) that preserve the progression of multiple biological processes, including long-term synaptic transmission, vessel contractility, glucose sensing, and pro-inflammatory setting, among others [39,40,41,42]. Moreover, the biology of Cxs encompasses another GJIC-independent function, i.e., to activate signaling pathways and affect cellular phenotypes by interaction of their C-terminal domain with partnering proteins. Interacting partners include β-catenin, Zo-1, v-SRC, PKC, cadherin, caveolin, MAPK, Skp2, and Bcl2 proteins, among the others [43,44]. Each of the three functions includes pro- or antitumorigenic roles for different connexin subunits (Figure 1).

## 2. GJ, GJIC, and Cancer

In 1966, Loewenstein and Kanno showed and described the loss of functional GJs in cancer cells that led to the hypothesis that GJIC was involved in the carcinogenesis process [45]. A number of promoter chemical and biological compounds, including toxins, organic solvents, pesticides, pharmaceuticals, peroxides, metals, and phthalates, are able to inhibit GJIC [13,46]. This drew the growing attention of researchers and led to focusing on the link between cell proliferation and GJIC paving the way to the proposal of novel pathogenetic models of cancer.

In 1989, Klann et al. demonstrated reduced GJIC in a panel of cell lines derived from selected stages of a mouse skin carcinogenesis model, using dye transfer methodology [47]. Then, Yamasaki et al. [48], investigating in vitro cell transformation and animal carcinogenesis models, suggested involvement of blocked intercellular communication in the early stages of carcinogenesis. In particular, they provided evidence indicating that aberrant GJIC was associated with the tumor promotion phase of carcinogenesis and with maintenance of transformed or tumorigenic, or both, phenotypes. These findings implied that not inhibited normal GJIC might accomplish a tumor-suppressive role. Intriguingly, they demonstrated by knocking out adhesion molecule genes that were unnecessary to directly alter GJ in order to block intercellular communication [48].

Additional studies tried to reveal a chemopreventive effect using antioxidant substances, such as all-trans-retinoic acid (ATRA), lycopene, curcumin, etc.; recently, Babica et al. [49] proposed a method to screen a chemical with potential chemopreventive activity based on GJIC measurements.

A number of subsequent studies further highlighted the role of Cxs and GJs in carcinogenesis and showed their involvement in numerous diseases, as well [43]. In particular, the loss of GJ or Cxs, or both, and its relationship with abnormal proliferation rates and absence of GJIC regulation in cancer has been demonstrated [25,50,51]. On the other hand, Cxs themselves have been found to promote tumor cell growth and invasiveness, contributing to the overall tumorigenicity. For example, Kar et al. [52] proposed that Cxs (Cx26, Cx37, and Cx43) might also be crucial in metastasis. Insightfully, they noted that intact GJs acted as tumor suppressors in the initial stages of tumorigenesis, controlling cell proliferation; indeed, inhibition of GJ resulted in uncontrolled proliferation (promotion), whereas in migrating tumor cells, re-expression of Cxs promoted tumor metastasis (metastasization). In 2017, Aasen et al. [25], reviewing 50 years of results, concluded that Cxs and GJIC can protect cells from cancer in earlier stages, but they can also induce it facilitating GJIC between cancer cells and the hosting environment, thereby providing support for aggressive late-stage tumors, as described by Osswald et al. [53] and Chen et al. [54]. Consistent with this notion is the rationalization of why Cxs increment in tumors is reported to be associated with either poor or better prognosis [25,55,56,57,58,59].

In 2018, Graham et al. [60] highlighted the need to better deepen the aforementioned contradictory data regarding Cxs. In particular, they noted that the involvement of Cxs in cell growth control is unclear at the molecular level and either appears to be GJIC-dependent or not. They questioned that Cxs could act as tumor suppressors as not fulfilling standard requirements. In the first place, Cx gene mutations have not been found in tumors, as commonly shown for p53, Rb, and other tumor suppressor genes [8]. Second, no clear mechanism of growth control has been established for Cxs contrary to what has been observed with classical tumor suppressors. Moreover, Cxs could play as both tumor suppressor and tumor enhancer. For example, Elzarrad et al. [61] noted that Cx43 plays an important role in the metastasis, particularly during intravasation and endothelial attachment when the communication between cancer cells and endothelial cells (EC) occurs. These apparent contradictory roles are likely due to the different expressions of Cxs: some tumor cells would be expected to proliferate (non-expressing Cx43), whereas others would be expected to migrate (Cx43-expressing cells) [62]. This could be an acquired ability that gives evidence of plasticity linked to homeostasis but, in any case, linked to cancer disease. Regarding the role of Cxs as tumor enhancer, it has been indicated that, in case of GJIC between cancer cells and cells of the tumor microenvironment (TME), Cxs may increase motility (such as glioma cells communicating with astrocytes) and, furthermore, promote intravasation and extravasation processes through GJIC-independent mechanisms mediated by the Cx C-terminal domain.

Pannexins are GJ-related proteins that have been involved in cell growth control, as well as in invasion and metastasis, even though their role is relatively unexplored compared to Cxs [60]. Considering the contradictory data on Cxs and the similarity of pannexins with Cx hemichannels, Graham et al. [60] suggested considering their involvement only in particular types of tumors, rather than globally. Finally, they proposed to verify the Cxs/pannexins ratio in the various cancer types at the same moment because it could permit to differentiate the opposite effects of Cxs.

In line with the aforementioned contradictory effects of GJIC in cancer development is the observation by Spray et al. [63], who described the so-called “Good Samaritan” and “Bystander” effects of GJIC by examining two opposite conditions where GJIC entailed a “kiss of life” or “kiss of death”. In particular, following exposure to toxic compounds, GJIC could either determine a dilution of the effect incrementing the effective volume available or, on other hand, spread cytotoxicity to adjacent cells. Then, they suggested the possibility of using these opposed effects as therapeutic possibilities.

**Figure 2:**
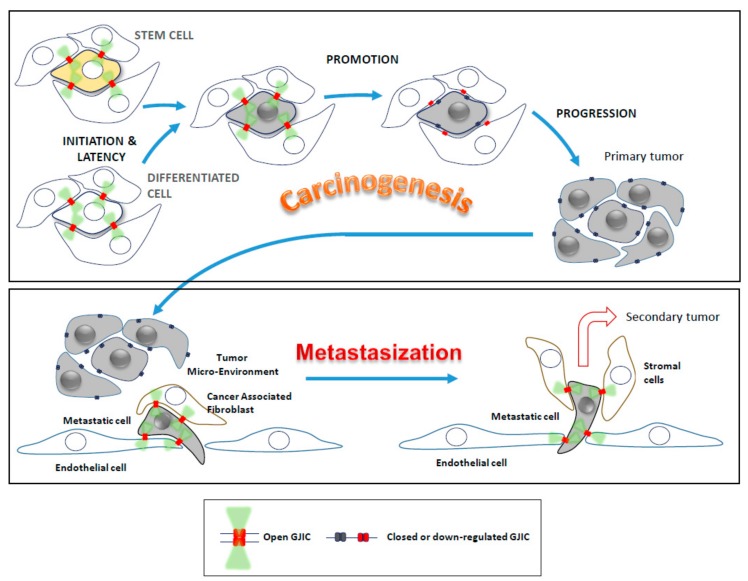
Different roles of GJIC in early and late phases of carcinogenesis involving either stem cells or differentiated cells. Intact (not inhibited) GJIC acts as tumor suppressor in the initial stages of tumorigenesis, it is inhibited in the promoter phase, and it is reactivated (re-opened) during the metastasization stage. In migrating tumor cells, re-expression of Cxs promote tumor metastasis, enabling cell-to-cell communication between tumor cells and cancer-associated fibroblasts, as well as endothelial and stromal cells. The same might happen in the secondary tumor.

Thus, GIJC between a circulating tumor and EC may represent a functional link between inflammatory processes and the local progression of primary tumors. The above concise survey demonstrates the multi-faceted and complex role of GJIC. Figure 2 synthetically shows these different roles that, at first glance, would appear contradictory.

### Carcinogenesis Models and Cancer Hallmarks

An important paper published by Vineis et al. [64], in reviewing the major current models of carcinogenesis, clustered them in five groups: mutational, genome instability, non-genotoxic, Darwinian, and tissue organization. However, the Darwinian model satisfactorily integrated all the others, highlighting the important role played by microenvironmental, genetic, and epigenetic factors. The same issue was addressed by Goodson, and numerous other authors [65], that constituted a non-profit association network of collaborating scientists to answer different questions regarding carcinogenesis, focusing on toxic effect and criticizing the classical toxicological approach to explain cancer disease in terms of risk assessment. The authors reviewed 85 examples of chemicals for actions on key pathways/mechanisms related to carcinogenesis; then Hanahan and Weinberg described the following six hallmarks of cancer [19]:

(1) Self-sufficiency in growth signals (later renamed proliferative signaling)—i.e., cancer cells grow at a seemingly unlimited rate.
(2) Insensitivity to antigrowth signals (evading growth suppressors)—i.e., cancer cells are not subject to antigrowth signals nor withdrawal of normal growth signals.
(3) Evading apoptosis (resisting cell death)—i.e., cancer cells avoid the usual process, whereby abnormal or redundant cells trigger internal self-destroying (as opposed to cell death) mechanisms.
(4) Limitless replicative potential (enabling replicative immortality)—i.e., cancer cells do not senesce (or age) and die after a limited number of cell divisions.
(5) Sustained angiogenesis (inducing angiogenesis)—i.e., cancer cells elicit new blood vessels to sustain growth.
(6) Tissue invasion and metastasis (activating invasion and metastasis)—i.e., in situ or non-invasive cancers grow into pre-existing spaces, but invasive tumors must create a space to expand into normal tissue.

Later, the same authors [66] expanded these hallmarks to encompass additional areas suggested by subsequent cancer research. They verified in the tumors the presence of further characteristics: i) genome instability which allows the accumulation of mutations and epigenetic changes that are passed from one cell to daughter cells; ii) tumor-promoting inflammation, which helps cancer cells to grow via the same growth signals that normal cells provide to each other during wound healing and embryonic growth; and iii) inflammation further contributes to the survival of malignant cells, angiogenesis, metastasis, and the subversion of adaptive immunity [67]. Considering these characteristics, the authors suggested adding two further cancer hallmarks: a) avoiding the immune system whereby tumor cells escape immune surveillance that would otherwise mark them for destruction; and b) dysregulated and highly adaptive metabolism.

**Figure 3:**
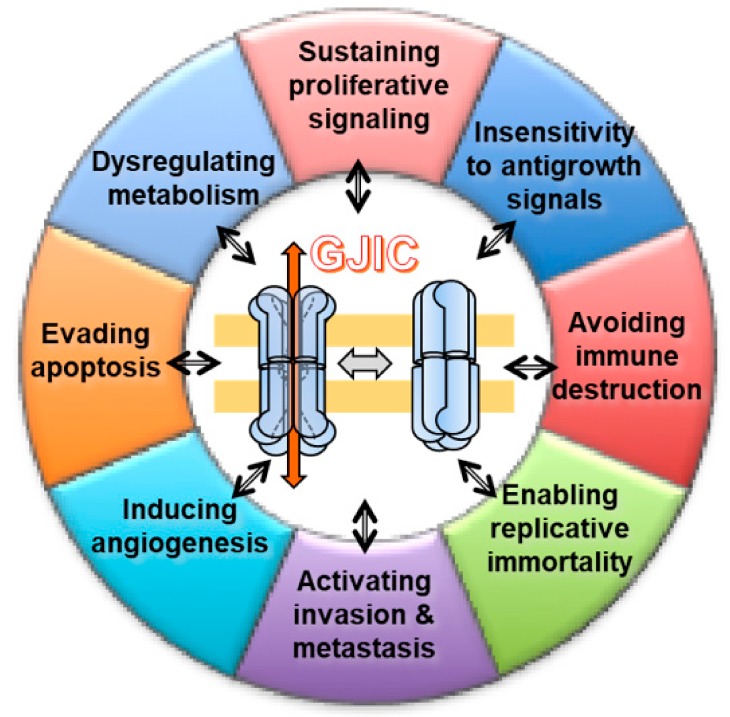
Schematic overview of the cancer hallmarks, as defined by Hanahan and Weinberg [66], and the involvement of GJIC in all of the hallmarks discussed in this review.

Taking into account all these hallmarks, we would consider and discuss the involvement of GJIC in each of them (Figure 3). If the hallmarks are acquired functional capabilities via distinct mechanisms and at various times during the course of multistep tumorigenesis, it could be helpful to identify how and when GJIC is involved.

## 3. GJIC and Hallmarks of Cancer

### 3.1. “Insensitivity to Antigrowth Signals” and “Sustaining Proliferative Signaling”

The role of GJIC in these hallmarks is indisputable; in fact, GJIC inhibition is a primary mechanism of action of promoter substances (Lindane, TPA) [68]) conferring insensitivity to growth-arresting signals. Regarding the circumstance of a tumor that evades growth suppressors, it could be useful to cite Nahta et al. [69], who showed that bisphenol A, a common component of plastic which exerts estrogenic effects, promotes cell cycle progression by disrupting multiple targets, including functional p53, determining dose-dependent evasion of apoptosis and increased proliferation [69,70]. In addition, low nanomolar concentrations of bisphenol A reduced expression of Cx43, compromising GJ communication [71]. Bisphenol A-mediated effects on GJIC are connexin-selective, as reduced expression of Cx43 has been observed after bisphenol A exposure, while Cx26 is unaffected [72]. Lindane, an organochlorine chemical used as an insecticide, was found to markedly suppress upregulation of the p53 damage-response pathway in bystander cells, as determined both by Western blotting and in situ immunofluorescence [73].

On the contrary, if we considered the hallmark “Sustaining proliferative signaling”, it will be useful to note that, during later stages, the functionality of GJIC permits cancer cells to communicate with other cells and then the tumor to progress (see above Osswald et al. [53] and Chen et al. [54]).

Overall, while the inhibition of cell–cell communications between an initiated cell and its contiguous neighbors has been shown in early studies concerning the tumor insensitivity to growth-arresting signals, further work has demonstrated that tumor progression does indeed depend on functional GJIC allowing communication with other cells.

### 3.2. Dysregulating Metabolism

Altered metabolism constitutes one of the earliest described hallmarks of cancer [74,75]. The so-called Warburg effect defines the propensity of cancer cells to favor metabolism via aerobic glycolysis, rather than the much more efficient mitochondrial oxidative phosphorylation pathway, which is the preference of most other cells of the body. Accordingly, how the cell rewires its metabolism during tumor transformation attracted the attention of investigators [76]. However, the paradigm of the Warburg effect in cancer has been recently challenged because a) not all types of tumors rely on aerobic glycolysis, and a number of them are characterized by oxidative metabolism; b) the metabolic phenotype of a given cancer cell can be rewired in the different stages of its development; and c) the specific cellular microenvironment of the tumor niche influences the metabolism of the cancer cell [77].

The TME forms a protumorigenic cocoon around the tumor cells, where reprogramming of the metabolism occurs in tumor and non-tumor cells, that underlies the nature of interactions, as well as competitions, ensuring a steady supply of nutrients and anaplerotic molecules for the tumor cells that fuels its growth, even under hypoxic conditions. This metabolic reprogramming also plays a significant role in suppression of immune attack on the tumor cells and in resistance to therapies. Thus, the metabolic cooperation and competition among the different TME components besides the inherent alterations in the tumor cells arising out of genetic, as well as epigenetic, changes supports growth, metastasis, and therapeutic resistance [78].

In a recent study, Luo et al. [79] investigated the role of Cx43 and its derived GJIC in the interplay between non-small cell lung cancer (NSCLC) cells and cancer-associated fibroblasts (CAFs). It was demonstrated that, in co-cultured cells, a metabolic coupling occurs between CAFs and NSCLC cells and that this relies largely on the formation of functional unidirectional heterocellular GJIC from CAFs to NSCLC cells. In particular, CAFs enhanced aerobic glycolysis, while NSCLC cells were shifted toward a mitochondrial oxidative metabolism, resulting in EMT/invasive/migratory effects. These effects were damped by inhibitors of Cx43 and glycolysis. Mechanistically, these findings can be explained in terms of enhanced transfer of oxidizable metabolites from CAFs (i.e., lactate, glutamine) to NSCLC cells via Cx43-mediated GJIC. The resulting increase in ATP production in NSCLC cells would be involved in activation of the PI3K/Akt and MAPK/ERK pathways and in providing bioenergetic support to promote the malignant behavior of NSCLC cells.

The aforementioned evidences strongly suggest that metabolic reprogramming toward a more mitochondrial oxidative phenotype might determine an escape from immune attack and resistance to therapies. This would be partly contributed by enhanced unidirectional heterocellular GJIC enabling acquisition of EMT/invasive/migratory ability.

### 3.3. Evading Apoptosis

This hallmark is another mechanism certainly controlled through GJIC. It is known that inhibition of GJIC enables the cells to evade apoptosis. This was demonstrated by Kameritsch et al. [80], who found that Cx43 and Cx40 promote apoptosis via GJ transfer of pro-apoptotic signal between cells (i.e., Ca^2+^, IP3) and that this was prevented by GJIC blockers. In addition, the non-channel-related function of Cxs has been suggested by the finding that cytoplasmic Cx26 co-localizes with Bcl-2 proteins, known effectors of the intrinsic apoptotic pathway [44,81].

In the context of apoptosis and, more generally speaking, of cell fate, mitochondria are a well-recognized decisional hub [82]. Following alterations of the mitochondrial membrane-related permeability, elicited by a number of both internal and external stimuli, pro-apoptotic factors are released in the cytosol which trigger the terminal caspases-cascade activation. It has long been known that tumor mitochondria are less sensitive to pro-apoptotic stressors, thus enhancing cancer cell survival [83]. In addition to their canonical localization at the plasma membrane, Cxs, in particular Cx43, have been identified at the outer mitochondrial membrane [84]. The functional role of the mitochondrial Cx43 is uncertain, though their protective function in reperfused cardiomyocyte has been demonstrated [85]. How Cxs are assembled in the mitochondrial membrane is an open issue. The occurrence of hemichannel connexons in individual mitochondria and the possibility to form GJ intracellular inter-mitochondrial communication is an attractive possibility that warrants further investigation. How the mitochondrial Cxs impact mitochondrial functions, dynamics, and control of cell fate in particular, in the context of tumorigenesis, is a completely open question at the moment.

### 3.4. Enabling Replicative Immortality

Cancer cells do not senesce (or age) and die after a limited number of cell divisions, as normal cells do. Ku et al. [86] showed that PKC controls telomerase activity. In fact, PKC inhibitors were shown to inhibit telomerase activity. Intriguingly, it is well known that PKC phosphorylates Cx43 and inhibits GJIC [87]. This points to a possible functional link between GJIC and telomerase activities in controlling cell senescence and immortality.

Yang et al. [88] showed that gastric epithelial cells with functional GJIC could acquire immortalization using N-methyl-N-nitro-N-nitrosoguanidine (MNNG), a chemical carcinogen able to activate telomerase. Since cells acquire easier immortalization if their GJIC is functional, this could indicate that this phase would be an initial priming condition because it would precede the inhibition of GJIC typical of the promoter phase.

An important contribution in the carcinogenesis discussion about limitless replicative potential was a paper published by Trosko et al. [89], who postulated that immortalization is not a real cancer hallmark because, if cancer arises from stem-like cells, it is naturally immortal. Indeed, Trosko [90] hypothesized a role for GJs in carcinogenesis, considering that adult stem cells do not express connexin genes and lack functional GJIC until they are induced to differentiate. Thus, the target cells for carcinogenesis are the adult stem cells, which are constitutively “immortal” until induced to express Cx genes and to differentiate [91,92,93,94,95,96]. If these stem cells, or their very early differentiated daughter cells, that have started to express their Cxs and to differentiate but have not yet down-regulated their telomerase activity, are exposed to a carcinogenic “initiator”, they will remain “immortal”; if expanded by mitogenic means or prevented from apoptotic death, they can accrue additional genetic and epigenetic changes to acquire the so-called “Hallmarks of Cancer”. Accordingly, it has been demonstrated that certain stem cells within a tumor were responsible for tumor progression, relapse, development of metastases, and resistance to therapies [97], and they are called cancer stem cells (CSCs), or “tumor-initiating cells” [98,99]. CSCs have the ability for self-renewal and differentiation, and they display roles similar to common stem cells in tissue development and regeneration [100]. The introduction of the “stem cell” hypothesis in carcinogenesis has mutated prospective from Trosko’s assumption to a more complicated picture by which connexin and GJs are protumorigenic and also that the simple hypothesis that GJs acts as a tumor suppressor fails to model the Cx diversity, driving communication rates in a cell-type-dependent manner [101]. This is highlighted by the fact that cancers are very heterogenous in having both CSC and non-CSC populations that are located on different ends of the differentiation spectrum. Recent evidences uncover a role for Cxs in CSC maintenance and self-renewal that has also been experimentally demonstrated in somatic stem cells during regeneration [102,103,104], in the germline [105], and in embryonic stem cells [106,107].

At the level of CSCs, many studies have found that some properties of CSCs, such as self-renewal, depend on GJIC in breast and hepatic cancers [108,109]. In addition, it has been found that GIJC was an integral part of CSC function also resulting in tumor progression [110,111]. Hitomi and colleagues demonstrated that GJs are present in glioblastoma CSCs and that Cx46 is essential for CSC proliferation, self-renewal, and tumor initiation in vivo, whereas Cx43 is expressed by non-CSCs [110]. On the other end, in glioma CSCs, it was found that down-regulation of Cx43 by hypermethylation of its promoter determined GJIC inhibition and that reconstitution of Cx43 inhibited CSC self-renewal, invasive capability, and tumorigenicity via a GJIC-independent manner through the regulation of the Wnt/catenin signaling pathway [112]. Likewise, it has been demonstrated in triple negative breast cancer that Cx26 expression was higher in CSCs and promotes self-renewal by forming a signaling complex with NANOG, a pluripotency transcription factor, and focal adhesion kinase (FAK), thereby again independently of GJIC [113]. These findings expand our concept of the classical view on Cxs function in CSCs and tumor initiation and progression. Overall, an emerging concept, i.e., that connexin set-up not only differs between cancer types but also displays regional or cell-type specific variations [34]. In summary, the protumorigenic actions of Cxs and GJIC are also dependent on their regulation of CSCs, which are now implicated in the many different phases of carcinogenesis.

### 3.5. Inducing Angiogenesis

Hanahan and Folkman [114] affirmed that, like normal tissues, tumors require sustenance in the form of nutrients and oxygen, as well as have an ability to evacuate metabolic wastes and carbon dioxide. EC are critically affected during the angiogenic process as their proliferation, motility, and morphology are modulated by proangiogenic and environmental factors associated with tumor tissues and cancer cells. Recent in vivo and in vitro studies have revealed that the GJs of EC also participate in the promotion of angiogenesis. Proangiogenic factors modulate GJ function and connexin expression in EC, whereas endothelial Cxs (mainly Cx37, Cx40, Cx43, as well as Cx32 and Cx45) [114,115,116] are involved in angiogenic tube formation and in the cell migration of EC. Several mechanisms, including GJ function-dependent or -independent pathways, have been proposed [115]. In particular, the Cxs reported above might have the potential to regulate cell mechanics, such as cell morphology, cell migration, and cellular stiffness, that are dynamically changed during the angiogenic processes.

Elzarrad et al. [61] showed that GJIC favored the interplay between cancer cells and EC, proving the involvement of GJIC in this crucial phase. In particular, they showed that adhesion of breast cancer cells to pulmonary endothelium correlated positively with Cx43 expression and, importantly, Cx43 expression increased in tumor–cell–EC contact area, suggesting that Cx43 can serve as a marker of tumor vasculogenesis. However, tumor cell Cxs appear to have a inhibitory role on tumor angiogenesis. For example, McLachlan and colleagues [117] found that the overexpression of Cx26 or Cx43 in breast cancer cells is associated with the upregulation and secretion of IL-6 and MCP-1, which in turn inhibit EC tube formation in vitro and tumor vascularization in vivo. Interestingly, the in vitro results were obtained in three-dimensional cultures (tumorspheres), which approaches the in vivo situation. Conversely, the silencing of Cx43 in breast cancer cells results in increased vascular endothelial growth factor (VEGF) expression and decreased thrombospondin expression [118]. It is worth noting that VEGF is known to regulate the expression of Cx43 and block GJIC [119,120]. Thus a complex interplay between Cx43-GJIC and VEGF is at work in tumors, and further studies are needed to comprehend the possible “epiphenomenon” nature of Cx43 acting as tumor suppressor. Other studies demonstrated that overexpression of Cx43 in melanoma and breast cancer cells suppresses tumor angiogenesis by decreasing transcriptional activity of HIF-1α and inhibiting the expression of VEGF [121]. Since a truncated Cx43 that could not form GJs inhibited the tumor growth [122], it is possible to infer that Cx43 inhibited tumor growth via a GJIC-independent mechanism [123]. Furthermore, the in vitro results correlated well with in vivo data, where it was shown that Cx43 knockdown increased vessel density in melanoma tumor models [121]. Overall, these findings indicate that Cxs are tumor suppressors via the inhibition of angiogenesis and that Cx43 may be a useful target for treating solid tumors by down-regulation of tumor angiogenesis.

The role of vascular Cxs has also been highlighted [124]. It has been shown that primary and metastatic tumor cells can modulate connexin expression in EC and favor a cross-talk between cancer cells and EC, promoting the transendothelial migration of malignant cells or neo-angiogenesis in the metastatic foci [125]. Similarly, glioblastoma multiforme cells can modulate Cx43 expression in EC by the GJ-mediated transfer of the miRNA mir-5096 promoting endothelial tubulogenesis [126]. Alonso and colleagues [116] have provided data on the involvement of EC Cx40 in tumor growth. Knockout mice which lack Cx40 presented lower tumor growth and angiogenesis than control wild-type mice. However, the inhibition of EC Cx40 promoted the recruitment of vascular SMCs and decreased eNOS activation in Cx40^-/-^ mice, implying the maturation of neo-vessels and resulting in an improved perfusion of the growing tumors. These data identify Cx40 as a potential novel target in cancer treatment.

Cxs and GJIC can have many different actions at the level of tumoral EC and neo-angiogenesis. Understanding this complexity will bring novel and more efficient anti-cancer therapies.

### 3.6. Activating Invasion and Metastasis

The TME is the stage where tumor cells, besides their intrinsic properties, are instructed to invade tissue and subsequently metastasize. In this regard, it is worthy to cite Chantraine et al. [127], stating that: “Self-sufficiency in growth signals, the ability to evade apoptosis, insensitivity to antigrowth signals, and limitless replicative potential are four among the six hallmarks that have their basis on genetic and epigenetic changes in the genome of the cancer cells. In contrast, sustained angiogenesis and the capability to invade and metastasize are two hallmarks that have their basis not only on genetic and epigenetic alterations but also on dramatic changes in normal cellular and acellular elements that support malignant cells. These non-malignant components of the tumor are known to form the TME. For the last two decades, the primary focus of investigations on the microenvironment has been on the tissues that are directly adjacent to primary and metastatic tumors”.

The heterotypic GJIC among cancer cells and stromal cells are indeed associated with protumoral properties [124]. First of all, GJ coupling between tumor cells and EC contributes to the invasion and metastasis. This has been demonstrated in melanoma by modulating Cx26 expression [128]. Cx26-expressing melanoma subclone BL6 cells established efficient coupling with EC, whereas CX26-negative subclone F10 does not. Interestingly, BL6 cells display a heightened metastatic potential compared with F10 cells. Others have shown that decreased Cx-43 expression after silencing of protease-activated receptor-1, a key player in melanoma metastasis, affects melanoma cell attachment to EC [129]. More recently, it was shown that metastatic 4T-1 breast cancer cells form functional GJs with EC via Cx43 and that Cx43 expression is required for metastatic breast cancer cell extravasation and blood vessel co-option in the brain, while metastatic melanoma B16 cells use Cx26 for brain perivascular microtumor formation [130].

An interesting picture is emerging about the communication between cancer cells and astrocytes and its role in tumor cell invasiveness in the brain. In 1999, Zhang et al. [131] reported GJIC-mediated direct cellular coupling between astrocytes and glioma cells that express mainly Cx43. This resulted in a phenotypic transformation of astrocytes that contributed to the susceptibility of surrounding tissue to glioma invasion. Following studies have confirmed that malignant glioma cells can form heterocellular GJIC with reactive astrocytes by using Cx43 and that this interaction is proficient for tumor cell invasion [132,133,134]. Czyz et al. [135] observed the effect of GJIC in later stages of tumorigenesis and showed that the expression of Cxs in blood and EC is modulated by pro-inflammatory agents, such as LPS and TNF-α [136], and that a switch from Cx37/Cx40 to Cx43 expression took place in EC during inflammation [137]. Thus, GJIC between the circulating tumor and EC may represent a functional link between inflammatory processes and the local progression of primary tumors.

Kar et al. [52] proposed that Cxs might also be crucial in metastasis. They noted, in the initial stages of tumorigenesis, that intact GJs acted as tumor suppressors, whereas in migrating tumor cells, re-expression of Cxs promoted tumor metastasis. Particularly, overexpression of Cx43 in human breast tumor cells at early stages, E9 mouse lung carcinoma, and osteosarcoma cells results in decreased cyclin D1 expression, hence acting as a tumor suppressor [122,138]. Conversely, Cx26 and Cx37 are expressed on the plasma membrane of cells invading the lymph nodes, even though their expression is reduced in the early stages of tumorigenesis [139]. Similar findings are reported in prostate [140,141], breast [142], and glioma [143] cancer cells. Furthermore, overexpression of Cx43 in HeLa cells increases their metastatic potential [144]. In melanoma cells, Cx26 level is unchanged in the basal layer; however, when these cells invade the dermis, the expression of this connexin is upregulated, and GJ is formed between the cancer cells and the endothelium [128,145].

Besides the primary tumor, the role of CAFs appears important also in the secondary site (metastatic). Through different methods and hypothesis, it was shown that the role of CAFs is essential to reprogramming not only metabolic profiles of cancer cells but also to increment tumor growth, invasion, and metastasis by inducing epithelial-mesenchymal transition (EMT) [146,147]. Indeed, Wang and colleagues found that CAFs induced lung metastasis and the EMT process in vivo by endometrial cancer cells [148]. There are evidences that CAFs overexpressing Cx43 support a mutual signaling between epithelial cells and stromal cells [149], promoting tumor development; the same role of CAFs might be envisioned in the metastatic foci (Figure 2).

No less important appears the involvement of EC and the formation of heterocellular GJIC with cancer cells for tumor extravasation and metastasization. Expression of Cx43 in GJIC-deficient mammary epithelial tumor cells allowed the formation of functional heterocellular GJs with microvascular EC increasing tumor cell diapedesis [150]. Overexpression of Cx43 in breast cancer cells facilitated their adhesion to pulmonary endothelium, while adhesion decreased with cells expressing dominant-negative Cx43. Furthermore, leukemic cells form heterotypic Cx43-mediated GJs with EC, allowing cancer cell migration and extravasation [151,152]. In this context, Graham et al. [60] highlighted the need to deepen the contradictory data regarding Cxs (i.e., tumor suppressors vs tumor enhancers) considering that Cxs could be able to favor metastasis through their involvement in the formation of invadopodia and secretion of proteases during invasion process. For example, HTLV-1 leukemic cells not only form CX43-GJIC with EC but also produce metalloproteinase (MMP)-2 and MMP-9, allowing tumor cells to invade EC by an angiogenesis-like mechanism [151].

The bone marrow, enriched in cells and growth factors, represents a unique microenvironment that has many relationships with tumor growth also when the tumor is not localized primarily in bone marrow. Tumor cells leave the primary tumor as they intravasate into the blood circulation and are actively recruited into the bone marrow microenvironment. Another observation demonstrated that when homed to the bone marrow, tumor cells disrupt the homeostatic balance between osteogenesis and osteolysis and create a microenvironment that favors their growth and survival [153]. The other way around, bone marrow-derived stem cells (BMDSCs) leave the bone marrow osteoblastic niche to enter the blood circulation and colonize the primary tumor where they contribute to the tumor vasculature and to an inflammatory reaction that deeply affects malignant transformation and progression [154,155]. BMDSCs can also colonize distant organs and form a pre-metastatic niche that will attract circulating tumor cells and promote the formation of macroscopic tumors. In the opinion of the authors, the role of VEGF is essential in stimulating angiogenesis and recruitment of VEGFR1^+^-BMDCs. On this issue, it could be noteworthy citing Li et al. [156], who demonstrated that GJIC was essential to differentiate endothelial progenitor cell through VEGF transfer in the cells through Cx43.

Since the formation of heterotypic GJIC among cancer cells and stromal cells induces protumoral effects, the understanding of GJIC between tumor cells and their surrounding stromal cells will be used in future to potentiate current cancer therapies via the modulation of Cxs and GJIC.

### 3.7. Avoiding Immune Destruction

This hallmark concerns the immune system role in resisting or eradicating formation and progression of incipient neoplasia, late-stage tumors, and micrometastasis. The long-standing theory of immune surveillance proposes that cells and tissues are constantly monitored by an ever-alert immune system and that such immune surveillance is responsible for recognizing and eliminating the vast majority of incipient cancer cells and tissue nascent tumors. According to this, solid tumors that do appear have somehow managed to avoid detection by the various arms of the immune system or have been able to limit the extent of immunological killing, thereby evading eradication. Concerning the role of connexin-mediated intercellular connections and control of tumor growth and progression, GJICs are involved in a complex modulation of the immune system in the context of cancer. A large number of evidences support the notion that Cxs expression on tumor cells may either promote or block cancer progression, depending on the type of cells engaged at the TME. In general, tumor GJIC with immune cells are associated with antitumoral effects, while tumor cell interactions with stromal cells are associated with antitumoral or protumoral effects, depending on the particular context where these interactions occur [124,157]. In the issue of antitumoral immune cells, for example, it is useful to cite Tittarelli et al. [158], who verified that Cx43, the main GJ protein of the immune system [159], accumulates at the contact zone of natural killer (NK) cells and dendritic cells (DCs) or at the interface between NK cells and target tumoral cells, facilitating DC-mediated NK cell activation and cytotoxic activity against tumor cells.

It is known that immune surveillance by NK cells can lead to tumor rejection and control of tumor dissemination [160]. DC–NK cell crosstalk is important for the activation of NK cells and can affect the magnitude and quality of the antitumor immune responses in vivo [161,162]. It is known that the reciprocal activation of NK cells and DCs is a cell contact–dependent process and mediated through the formation of a functional immunological synapse (IS) [163]. Moreover, NK-mediated cytotoxicity of target cells largely relies on the formation of functional IS [164]. Once an IS is formed, NK cells can induce apoptosis in the target cells by releasing their cytotoxic granules, including granzyme B (GrB) [165]. Importantly, Ca^2+^ influx in target cells is required for the effective internalization of perforin and GrB and for immune-mediated death by apoptosis. This observation would have to be studied in depth, but, in our opinion, it is important for this hallmark. Concerning the heterotypic GJIC among cancer cells and stromal cells associated with antitumoral effects, many interactions have been described that bring this unfavorable outcome for the tumor, namely with EC [166], BMSCs [167], osteocytes [168], and fibroblasts during suicide gene therapy associated with the “bystander effect” [169]. The protumoral effects of heterotypic GJIC among cancer cells and stromal cells has been described in the previous Sections.

Although the immune escape of tumoral cells is a well-known phenomenon, to our knowledge no data have been published on the role of Cxs and GJIC in the immunosuppression. Since the involvement of Cxs in immune tolerance and Treg cell activity have been proven [124], this field of investigation is new and should be approached and pursued. It is likely that the tumor microenvironment plays an important role. This is evidenced, for example, by Tittarelli et al. [170], who showed that activation of autophagy in hypoxic melanoma cells causes the selective degradation of Cx43-GJIC impairing NK cell-mediated tumor lysis. This is a new area of investigation that could also pave the way for novel therapeutics against cancer.

**Figure 4:**
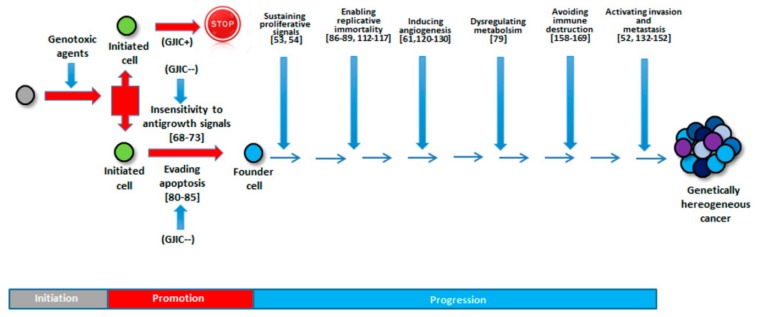
Involvement of GJIC in the different steps of carcinogenesis. Carcinogenesis is characterized by “initiation,” “promotion,” and “progression” phases. After a stem/progenitor cell is initiated by genotoxic agents, this is followed by promotion of cell growth. If functional GJIC is expressed, the initiated cell will be stopped at first step (Initiation). On the other hand, if these initiated cells are exposed, chronically, to agents that down regulate GJIC, they will lose sensitivity to antigrowth signals and evade apoptosis, giving rise to the so-called “founder” cell. During the progression phase, these cells would proliferate, accumulate, and accrue sufficient genetic/epigenetic changes that will allow them to acquire all the hallmarks of cancer. At the end, a tumor will be a mass of genetically heterogeneous cells. References showing the role of GJIC during all the described phases are indicated in square brackets. GJIC^+^: Gap Junction Intercellular Communication functioning; GJIC^--^: Gap Junction Intercellular Communication inhibited.

A synopsis of the above discussed issues is presented schematically in Figure 4.

## 4. Conclusions

In this review, we focused the attention on the contribution of Cxs and GJIC to all the hallmarks of cancer. If it is unquestionable that this contribution is real, nevertheless, their role appears different and differently deepened, proved, and investigated when we consider the various cancer hallmarks. All in all, defective GJs are either a prerequisite for or a consequence of cancer development; thus, GJIC could always act as an epiphenomenon that occurs alongside an event. Nevertheless, acknowledgement of the GJIC role could help in cancer prevention or even in the therapeutic control of the disease. This concept is testified by the fact that multiple mechanisms are likely to contribute to GJIC action in cancer. Indeed, in solid tumors, the promotion stage is linked to the loss of GJIC function, whereas the metastasis stage requires functioning GJs. In this regard, in order to improve the preventive arsenal, it might be useful to identify: i) screening methods assessing GJIC to reveal the promoter capability of compounds/condition and ii) chemopreventive drugs able to restore the inhibited GJIC. To note, several therapeutical agents are known to disrupt GJIC and hemichannel function, including those approved by the FDA for unrelated conditions (reviewed by Salameh and Dhein [171]), and that possibly exert their action during the invasion/metastatic stage. Due to the GJ pan-inhibition of these agents (e.g., carbenoxolone), having side effects involving normal cells, it will be necessary to develop peptides that inhibit specific Cxs expressed only in or in higher amounts in cancer cells [172]. Novel therapeutic approaches should also target hemichannel activity or Cx–protein interactions. Finally, given the possibility of existence of both CSCs and cancer transit cells, the treatment strategy would be either (a) to induce GJIC or, in the other case, (b) to reverse the posttranslational modification of the Cx protein, in order to restore normal GJIC [173]. However, recent results point out to the role of Cxs and GJIC in the self-renewal and other properties of CSCs, indicating that the intervention on these “initiated” cells is more complex than before envisioned. Furthermore, although in the case of true “embryonic” CSCs, it has been suggested that Oct4A gene and Cx genes are interrelated [174], these results should be confirmed since drugs that could transcriptionally repress the OCT4A gene may be beneficial in this context [174]. Due to multiple roles of GJIC in cancers, targeting intercellular communication in these devastating and lethally diseases is a promising area of development.

Vineis et al. [64] affirmed: “Science moves forward when a new theory emerges that explains not only previously unexplained findings but also all of the phenomena already explained”. In regard to this assertion, we assume that the focus on GJIC could permit to deepen cancer knowledge by integrating mechanisms, functions, and events that seemingly are separated from one another. In this regard, it might not matter if GJIC may appear as a phenomenon or an epiphenomenon because it is probably both; it all depends on our point of view, but above all, it depends on our intention to go further not counting only on it but by engaging to find other mechanisms, events, or functions that share GJIC; this could allow for a better understanding of carcinogenesis so that, hopefully, one day, all aspects could appear more clearly.
